# Supplementary material for: The flow experience: Polish adaptation and validation of the psychological flow scale (PFS)
Source: PLoS One. 2025 Dec 5;20(12):e0335907. doi: 10.1371/journal.pone.0335907 (PMC12680258; doi:10.1371/journal.pone.0335907)
Supplement: S1 Table — Table lists each item’s original (English) wording and Polish translation. (DOCX) [file pone.0335907.s001.docx]

| Supplementary Table S1. Bilingual item list for the Psychological Flow Scale (original English wording and Polish translation) | | |
| --- | --- | --- |
| Item no. | **English version (original)** | **Polish version (translation)** |
| 1. | I was absorbed in the act/task | Byłam/byłem pochłonięty działaniem/zadaniem. |
| 2. | I was highly focused on the task/activity | Byłam/byłem bardzo skupiony na zadaniu. |
| 3. | All my attention was on the task/activity | Cała moja uwaga była poświęcona zadaniu/aktywności. |
| 4. | I felt like I could easily control what I was doing | Miałam/miałem poczucie, że z łatwością mogłem kontrolować to, co robiłem. |
| 5. | My actions flowed effortlessly | Moje działania przebiegały bez wysiłku. |
| 6. | There was a sense of fluidity to my actions | Miałam/miałem poczucie płynności moich działań. |
| 7. | I found the experience rewarding | Uznałam/uznałem to doświadczenie za nagradzające. |
| 8. | The experience felt satisfying | To doświadczenie dało mi poczucie satysfakcji. |
| 9. | I would like the feeling of that experience again | Chciałabym/chciałbym ponownie poczuć to, czego wtedy doświadczyłem. |
