# Supplementary figures and images for: The flow experience: Polish adaptation and validation of the psychological flow scale (PFS)
Source: PLoS One. 2025 Dec 5;20(12):e0335907. doi: 10.1371/journal.pone.0335907 (PMC12680258; doi:10.1371/journal.pone.0335907)

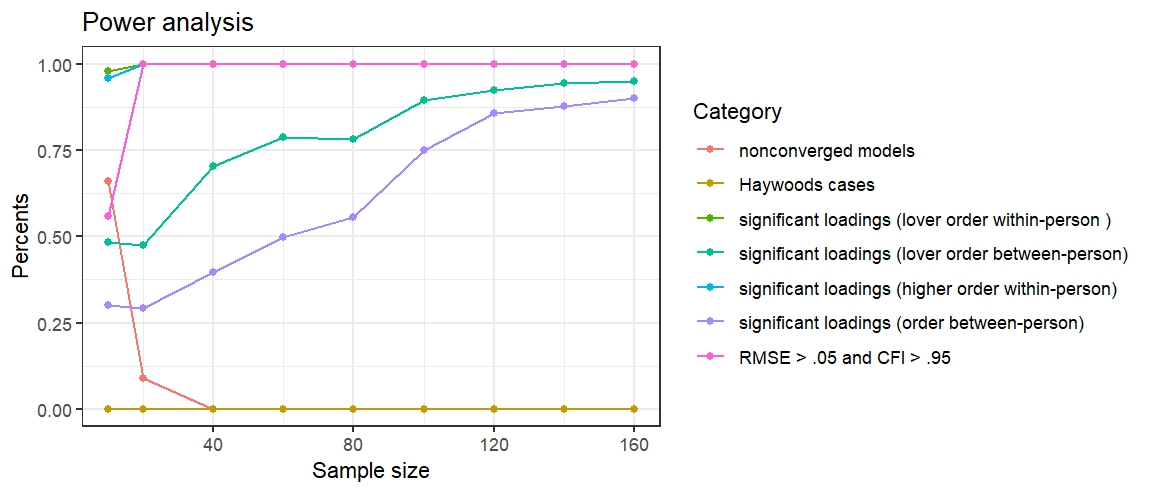

Supplement: S1 Fig — Graphical summary of simulation-based power for the key analyses reported in the manuscript. (JPEG) [file pone.0335907.s002.jpeg]
